# Next-Generation Harvester Technologies: Synergizing Smart Grading and Biomechanical Damage Control in Mechanized Tomato Production

**DOI:** 10.3390/s26103123

**Published:** 2026-05-15

**Authors:** Jianpeng Jing, Yuxuan Chen, Pengda Zhao, Bin Li, Shiguo Wang, Yang Liu, Zhong Tang

**Affiliations:** 1 College of Agricultural Engineering, Jiangsu University, Zhenjiang 212013, Chinazht@ujs.edu.cn (Z.T.); 2 Xinjiang Swallow Modern Agricultural Machinery Equipment Co., Ltd., Shihezi 832000, China; 3 Xinjiang Academy of Agricultural and Reclamation Science, Shihezi 832000, China20192309011@stu.shzu.edu.cn (S.W.);

**Keywords:** paste-type tomato, smart grading systems, multi-source information synergy, harvest-induced trauma, compliant handling mechanisms

## Abstract

Mechanized harvesting in the industrial tomato sector is currently bottlenecked by excessive mechanical injuries and elevated levels of foreign materials generated during electro-mechanical combine harvesting operations. To combat these limitations, this comprehensive review explores recent breakthroughs in harvester-mounted smart grading systems engineered specifically for complex, open-field conditions. Rather than relying solely on conventional optical inspection, the study examines the transition toward advanced, heterogeneous edge-computing frameworks—incorporating FPGAs and embedded GPUs—deployed within electro-mechanical harvesting platforms. This architectural evolution plays a crucial role in mitigating unpredictable processing delays caused by intense operational vibrations, although achieving absolute real-time stability under extreme field conditions remains an ongoing challenge. To minimize bruising and physical deterioration, our analysis synthesizes findings from multi-scale explicit dynamic finite element simulations, unpacking the underlying microstructural failure modes of the crop. We illustrate how regulating applied forces via soft robotic effectors can help approach a ‘damage-free’ handling threshold, though empirical results vary depending on fruit maturity and dynamic operational speeds. Furthermore, coupling multi-modal sensor fusion with Convolutional Neural Networks (CNNs) shows promising potential for non-destructive internal property evaluation under the vibration, dust, and throughput constraints of electro-mechanical harvesters, pending broader validation across diverse field datasets. Ultimately, by projecting future trends in onboard electro-mechanical harvester separation and advocating for a closer synergy between agronomic practices and machine engineering, this paper delivers a comprehensive blueprint for building next-generation, highly resilient, and gentle sorting machinery.

## 1. Introduction

**Figure 1:**
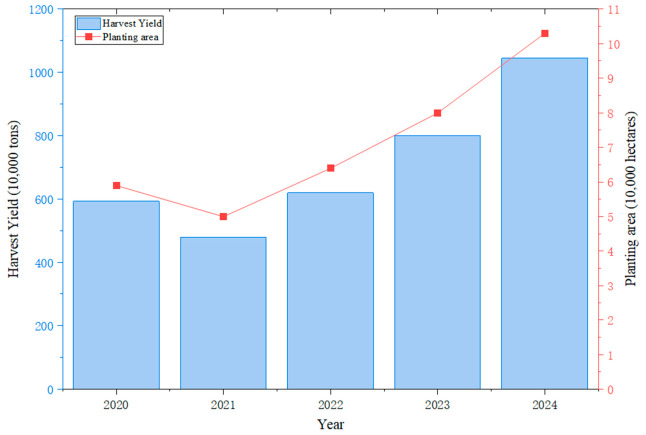
Planting area and production of processing tomato in China. Data presented in the figure were derived from the China Xinjiang Tomato Industry Association and World processing Tomato Council (WPTC).

The tomato ranks among the most significant fruit and vegetable cash crops globally, with its deep-processed products occupying a core position in international trade [1,2,3,4,5,6]. Due to favorable climatic conditions—such as extended sunshine duration and significant diurnal temperature variations—Xinjiang has emerged as a major processing tomato production region in Asia, contributing substantially to total domestic output. [7,8,9]. As shown in Figure 1, between 2020 and 2024, the domestic production of processing tomatoes increased from 5.94 million tons to 10.45 million tons, with the region accounting for majority of the increase. With the intensive expansion of planting scales and rising labor costs, electro-mechanical combined harvesting has become increasingly essential for maintaining the productivity of this industry [10,11,12]. However, while existing mechanized operation modes have significantly increased harvesting efficiency, they have also introduced a severe challenge of high impurity rates in raw materials due to non-selective one-pass harvesting mechanisms. These foreign materials and defective fruits not only seriously compromise the color and flavor of subsequent processing products like tomato paste but also directly threaten food safety, thereby constituting a critical technical bottleneck currently restricting the high-quality development of the processing tomato industry.

**Figure 2:**
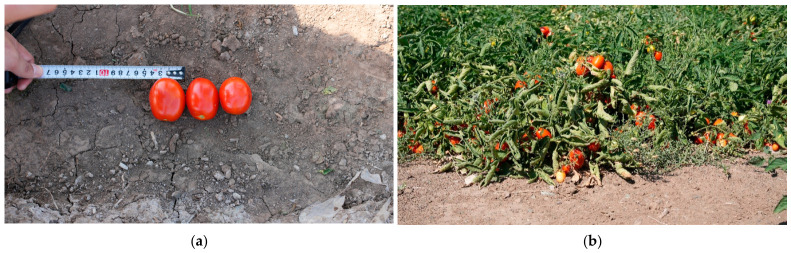
Fruit morphological characteristics and field growth environment of processing tomatoes. (**a**) Characteristics of processing tomatoes; (**b**) field distribution of processing tomatoes.

**Figure 3:**
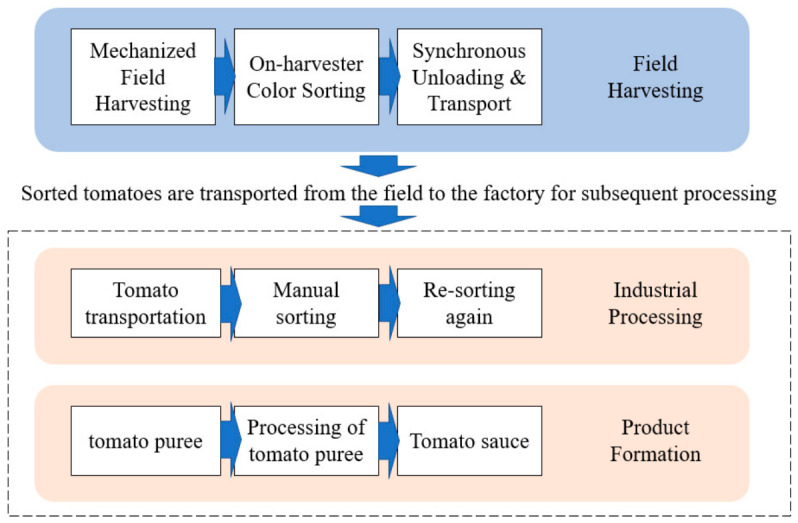
Schematic diagram of full-chain production process of processing tomatoes: from mechanized field harvesting to industrial processing and product formation.

As illustrated in Figure 2, since combine harvesters predominately adopt a non-selective, one-pass harvesting method, the harvested material flow is frequently mixed with substantial quantities of impurities. The typical production process of processing tomatoes involves a continuous transition from electro-mechanical field harvesting to factory-level deep processing. In this chain, the fruit sorting stage serves as a key node connecting electro-mechanical field harvesting and factory processing, directly determining the final product’s market value and consumer satisfaction [13,14,15,16]. In the initial stages of industrial development, raw material sorting relied primarily on manual operations. While this traditional mode of operation is intuitive, its drawbacks have become increasingly apparent when facing large-scale industrial production. First and foremost, the issue of low efficiency in manual sorting is particularly prominent. Especially during the peak tomato harvest season, there is a significant contradiction between the massive demand for raw material throughput and limited manual processing capabilities; this easily forms a bottleneck in the production process, seriously restricting the operational efficiency of the entire supply chain [17,18,19,20,21,22]. Secondly, with socio-economic development and changes in labor structure, labor costs have shown a rising trend year by year, and the traditional sorting mode, which relies on labor-intensive practices, has led to a substantial increase in production costs. For agricultural product processing enterprises with inherently limited profit margins, high labor expenses have become a heavy burden, limiting the expansion of their production scale and the improvement in their market competitiveness. As illustrated in Figure 3, the typical production lifecycle of processing tomatoes constitutes a continuous industrial chain spanning from the agricultural field to the processing plant. The process initiates with mechanized field harvesting, where combine harvesters often perform preliminary on-harvester color sorting to remove bulk impurities. Subsequently, the raw materials are transported via synchronous unloading to the factory for deep industrial processing. This stage involves rigorous cleaning, secondary refined sorting, and crushing, ultimately culminating in product formation (e.g., tomato paste). Within this workflow, the sorting technology acts as the critical quality gatekeeper connecting agricultural harvesting with industrial manufacturing. Notably, defects and impurities introduced during the harvesting stage cannot be fully eliminated by downstream factory-based sorting, underscoring the necessity for effective onboard sorting during electro-mechanical harvesting.

In the face of the aforementioned limitations, and with the advancement of agricultural modernization and intelligent manufacturing, the introduction of intelligent color sorting technology has become an urgent requirement for the transformation and upgrading of the tomato processing industry [23,24,25,26,27,28]. Based on advanced optical sensing and image processing algorithms, intelligent color sorting technology can effectively overcome the limitations of human vision to perform rapid and precise quantitative identification of tomato color, shape, texture, and even internal quality [29,30,31,32,33,34,35,36,37,38,39,40,41,42,43,44,45,46,47]. This technology enables all-weather, high-throughput automated operations, effectively overcoming the efficiency bottlenecks of manual sorting. Furthermore, establishing strict digital standards ensures objective and consistent sorting results, which substantially improves accuracy and yield [48,49,50,51,52,53,54]. Therefore, the development and application of efficient and precise intelligent color sorting equipment provide a viable approach to reduce labor costs and improve overall processing efficiency in large-scale agricultural production.

The development of sorting technology has consistently paralleled the expansion of agricultural production scale and the escalation of quality requirements, generally undergoing an evolutionary process from mechanized photoelectric sorting to intelligent multi-modal fusion. In the initial stages of industrial development, early optical color sorters emerged; these devices primarily utilized photodiodes to detect differences in the reflected light intensity of materials, achieving automated removal of heterochromatic particles based on single wavelengths or simple color difference principles. However, the capability of such equipment to identify defects with similar colors or complex shapes was relatively limited, making it difficult to meet the demands of refined sorting. Subsequently, the introduction of Charge-Coupled Device technology brought about a qualitative leap in detection accuracy. High-resolution color CCD sensors, coupled with image processing technology, could not only identify subtle color differences but also comprehensively assess material shape and texture; this significantly improved detection resolution from 2 mm^2^ of traditional photoelectric sorters to 0.082 mm^2^, establishing it as the mainstream technical method for industrial sorting for an extended period. Despite enhancing apparent detection capabilities, this technology still possessed limitations regarding the processing of complex field backgrounds and the detection of internal quality. Currently, sorting technology is entering a new phase characterized by intelligence and multi-modal fusion, with increasing emphasis on compatibility with electro-mechanical harvesting environments. With the deep integration of artificial intelligence and deep learning technologies, modern color sorting equipment is no longer limited to the shallow recognition of apparent features. Visual algorithms based on Convolutional Neural Networks can effectively extract high-dimensional features, solving challenges such as fruit overlapping, occlusion, and interference from complex environments. Crucially, the trend is shifting from single optical imaging toward multi-modal sensor synergy. While laboratory studies suggest that integrating hyperspectral imaging with tactile sensors significantly reduces fruit damage under controlled conditions, its high computational demand and hardware cost currently limit its transition to real-time deployment on electro-mechanical harvesters. Moving forward, this remains a promising but challenging future engineering vision.

Grounded in the context of intensive and large-scale production in the Xinjiang processing tomato industry, and addressing the critical industry pain points of high impurity rates and susceptibility to mechanical damage following mechanized harvesting, this paper comprehensively reviews the latest research progress and current status. This review is structured to progress systematically from theoretical foundations to key technologies, system integration, and future prospects. The objective is to synthesize current knowledge and offer technical insights for developing efficient, low-damage intelligent sorting systems.

To enhance the scientific rigor and reproducibility of this review, a literature search and screening protocol was established following the principles of the Preferred Reporting Items for Systematic Reviews and Meta-Analyses (PRISMA) framework. A comprehensive literature retrieval was conducted exclusively using the Web of Science (WoS) Core Collection, targeting English-language, peer-reviewed publications within a defined time span. The search was performed in the Topic (TS) field, covering titles, abstracts, author keywords, and Keywords Plus.

The search strategy employed Boolean operators to integrate core research concepts using queries such as (tomato OR “processing tomato“ OR “industrial tomato“) OR (sorting OR grading OR separation OR classification OR “foreign material removal“) AND (“electro-mechanical” OR electromechanical OR “control system”). This strategy was designed to ensure comprehensive coverage of studies relevant to electro-mechanical harvesting and harvester-mounted sorting technologies.

To minimize selection bias, explicit inclusion and exclusion criteria were applied. Articles were included if they focused on electro-mechanical technologies, sensing and sorting mechanisms, control strategies, or biomechanical damage control in tomato harvesting or onboard sorting systems. Studies were excluded if they primarily addressed agronomy, plant genetics, or factory-based post-harvest processing, or if they lacked sufficient technical detail, empirical validation, or accessible full texts.

**Figure 4:**
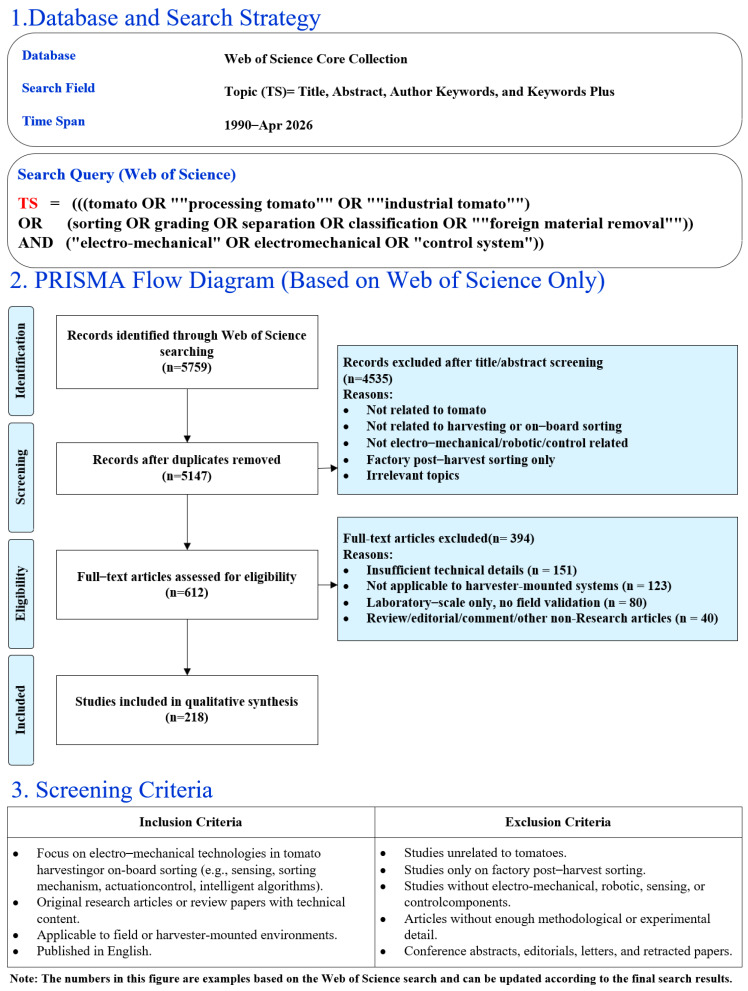
PRISMA flow diagram illustrating literature identification, screening, eligibility assessment, and inclusion process based exclusively on the Web of Science Core Collection for this review.

The screening procedure followed a stepwise process. After the initial retrieval, duplicate records were removed. The remaining studies underwent title and abstract screening to exclude publications not aligned with the scope of electro-mechanical harvesting. Subsequently, full-text articles were assessed for eligibility against the predefined criteria. Studies that satisfied all inclusion requirements were ultimately retained for qualitative synthesis to characterize the current technological landscape. The complete screening and selection process is summarized quantitatively as shown in Figure 4.

Chapter 2 deeply analyzes the material properties and operational environment challenges of processing tomatoes, which form the physical basis for achieving precise sorting. This section focuses on a comparative analysis of spectral reflection differences in visible and near-infrared bands among typical foreign objects such as red fruits, green fruits, moldy fruits, and soil clods; furthermore, based on a biomechanical perspective, it elucidates the damage thresholds and Hertzian contact stress response mechanisms of tomato fruits as viscoelastic bodies while also pointing out interference on visual perception caused by high dust levels, variable lighting, and material overlapping in unstructured field environments.

Building on this, Chapter 3 focuses on the system architecture and hardware selection of color sorting equipment. This chapter details the synergistic working principles of four major modules—feeding and conveying, photoelectric detection, signal processing, and sorting actuation—and, addressing the rolling characteristic of tomato spheres, compares the applicability of belt-type versus chute-type conveying devices; simultaneously, it discusses selection strategies for imaging hardware such as high-frequency LED light sources, high-speed color sensors, and multispectral cameras as well as the differences in dynamic response between air-jet and mechanical finger-type actuators.

Chapter 4 places emphasis on the evolutionary history of and latest breakthroughs in visual recognition algorithms. This section not only reviews traditional image processing methods based on color space conversion and morphological feature extraction but also deeply explores the application of deep learning technologies, represented by Convolutional Neural Networks, in tomato object detection and instance segmentation; particular attention is paid to the advantages of YOLO series algorithms in resolving fruit overlapping, occlusion, and complex background interference as well as the critical role of model lightweighting technologies in industrial deployment.

Addressing the specific characteristic that processing tomatoes have thin skins and are easily damaged, Chapter 5 specifically investigates low-damage sorting mechanisms and the design of flexible actuators. This chapter analyzes fruit damage forms under static compression and dynamic collision, highlighting the application of soft robotics technology and viscoelastic flexible materials in end-effector optimization.

Chapter 6 synthesizes the key challenges and future development directions of electro-mechanical harvester-mounted sorting systems, highlighting that multi-modal information fusion, in situ field sorting, intelligent adaptive control, and the integration of agricultural machinery with agronomy will be the core directions promoting industrial technology upgrades.

Through the aforementioned progressive review analysis, this paper provides comprehensive technical references for the research and development of electro-mechanical harvester-mounted color sorting equipment for processing tomatoes with independent intellectual property rights.

## 2. Material Characteristics and Sorting Challenges of Processing Tomatoes

### 2.1. Optical and Spectral Characteristics

As shown in Table 1, the optical absorption and reflection characteristics exhibited by tomato fruits and their associated impurities under irradiation by electromagnetic waves of different bands serve as the physical foundation for achieving intelligent and precise sorting [43,55]. In the visible light spectrum, specifically within the range of 380 nm to 780 nm, the reflectance of an object is primarily determined by its surface color and texture characteristics. Due to the substantial accumulation of carotenoids such as lycopene, ripe processing tomatoes exhibit high reflectance in the red spectral region, whereas their reflectance is lower in the green and blue regions. Conversely, immature green fruits possess an epidermis rich in chlorophyll, resulting in their spectral reflectance curves peaking in the green band. However, research indicates that the stems and leaves of tomato plants are also rich in chlorophyll, and their spectral reflection characteristics in the visible band are extremely similar to those of green fruits; this spectral overlap phenomenon makes it difficult for machine vision systems relying solely on visible light images to effectively separate green fruits from stem and leaf impurities against complex backgrounds. Furthermore, red soil clods or red stones in the field harvesting environment are similar in hue to ripe tomatoes, presenting a metameric phenomenon that further increases the difficulty of rejection based on color features.

To overcome the limitations of the visible light band, the introduction of the near-infrared (NIR) spectral band has become a key means of distinguishing between different materials [56,57,58,59]. NIR spectroscopy is mainly based on the overtone and combination absorption characteristics of specific wavelengths of infrared light by hydrogen-containing groups in organic molecules, such as C-H and O-H bonds [60,61,62]. As biological entities with high water content, normal tomato fruits strongly absorb NIR light due to internal moisture, thus exhibiting lower reflectance in the corresponding water absorption bands [63,64,65]. In contrast, impurities such as soil clods, stones, and dried straw have extremely low moisture content and typically exhibit higher reflection intensity in the NIR band, thereby creating a significant grayscale difference [66]. Regarding moldy fruits, fungal infection causes the disintegration of cellular structures, leakage of internal tissue fluids, and changes in chemical composition, leading to significant deviations in their scattering and absorption characteristics for NIR light compared to healthy fruits. Simultaneously, NIR light possesses a certain penetration capability, enabling the detection of tissue conditions beneath the fruit epidermis [67,68]. Therefore, combining color information from the visible light band with material information from the NIR band to construct a multispectral or hyperspectral feature space serves as the theoretical basis for achieving precise classification of red fruits, green fruits, moldy fruits, and various abiotic impurities [69,70].

**Table 1:** Comparison of physical and optical properties between processing tomatoes and typical rejects.

Material Category	Typical Component	Visual Color Characteristics	Spectral Response	Sorting Challenges
Acceptable Product	Mature Red Tomato	Red color. Exhibits high reflectance in the 600–700 nm waveband. The surface is smooth and glossy. [71]	Shows high absorbance in wavebands such as 970 nm and 1450 nm. Internal tissue is dense with specific light transmittance properties.	Serves as the benchmark target for sorting.
Defective Fruit	Immature Green Tomato	Green color. Exhibits a reflectance peak in the 500–550 nm waveband. Color is close to that of stems and leaves. [72]	The spectral curve highly overlaps with that of leaves. Moisture absorption characteristics are similar to those of red tomatoes.	Requires specific wavebands, such as the red-edge position, to distinguish minute differences from leaves.
Defective Fruit	Rotten Fruit	Discolored spots or darkening. Gray, black, or white mycelium may appear on the surface. Localized rough texture. [73,74]	Cell structure disintegration leads to changes in light scattering properties. Changes in internal chemical composition cause spectral shifts.	Early internal mold is invisible under visible light and requires NIR transmission detection.
Inorganic Impurity	Soil Clod	Red or brown color. Exhibits a severe “metamerism” phenomenon with mature red tomatoes. The surface is rough and matte. [75]	Extremely low moisture content; reflectance in the near-infrared moisture waveband is far higher than that of tomatoes.	Visible light cameras struggle to distinguish “red soil” from “red fruit”; detection must rely on NIR or texture features.
Inorganic Impurity	Stone	Gray, white, or red color. Colors are diverse and may be similar to the fruit. [76]	Spectral response is flat with no biological characteristic peaks.	Density difference is the core criterion; X-ray transmission rejection is the most thorough method.
Organic Impurity	Stem/Leaf/Vine	Green color. Rich in chlorophyll; color is consistent with green tomatoes.	Moisture content is lower than in fruit, with distinct cellulose absorption peaks.	Prone to entanglement and color interferes with green fruit sorting; primarily relies on morphological recognition.

### 2.2. Operational Environment Challenges

Integrating intelligent color sorting equipment directly onto large tomato combine harvesters for in-field in situ sorting represents a technological trend extending from post-harvest processing to the harvesting stage; however, this mobile operation mode faces more severe unstructured environmental challenges than fixed factory scenarios. First, intense and frequent mechanical vibration is the primary challenge faced by onboard color sorting systems. The surface shock generated when the combine harvester travels on undulating field terrain, superimposed with the high-frequency vibration from the engine and threshing drum, is directly transmitted to the color sorting module. This complex vibration environment not only causes focal drift and image motion blur in the optical imaging system, reducing edge detection accuracy, but also severely interferes with the stability of the material’s movement trajectory on the conveyor belt or chute, making it difficult for the airflow or fingers of the rejection mechanism to accurately hit the high-speed moving target fruit, thereby significantly reducing the sorting accuracy [77,78,79,80].

Secondly, high concentrations of airborne dust and a harsh polluted environment pose a significant threat to the reliability of precision optical systems [81,82,83,84]. During in-field in situ harvesting, the header and separation mechanism raise large amounts of dry soil particles and plant debris, forming a high-density dust aerosol environment. These particles easily adhere to the surfaces of lens glass and sensors, causing reduced light transmittance and increased imaging noise, which leads to a sharp decline in the feature extraction capability of recognition algorithms as operation time progresses. At the same time, the surface of freshly harvested tomatoes is often covered with wet soil, slurry, and plant juice [85,86,87]; these pollutants mask the original spectral characteristics of the fruit, making color-based recognition models prone to failure when distinguishing between red soil clods and dirty red fruit, resulting in severe metamerism interference.

Furthermore, dynamic and uncontrollable natural lighting conditions and unstable material flow states are also unavoidable challenges for onboard operations. Although onboard equipment is usually equipped with light shields and supplementary light sources, under intense direct outdoor sunlight or variable cloud cover, stray light may still enter the detection area through the inlet and outlet, disrupting the uniformity of the light field and causing image overexposure or shadows. More critically, the material flow directly from the harvester header possesses extremely high impurity content and flow fluctuations; large amounts of mixed hard stones, large soil clods, and entangled vines not only cause physical damage to flexible rejection mechanisms but also create detection blind spots due to high-density stacking and occlusion effects, making it difficult for traditional single-view vision systems to capture the defect features of occluded fruits, leading to a significant rise in the missed sorting rate.

## 3. Architecture and Key Hardware Technologies of the Color Sorting System

### 3.1. Overall System Architecture

**Figure 5:**
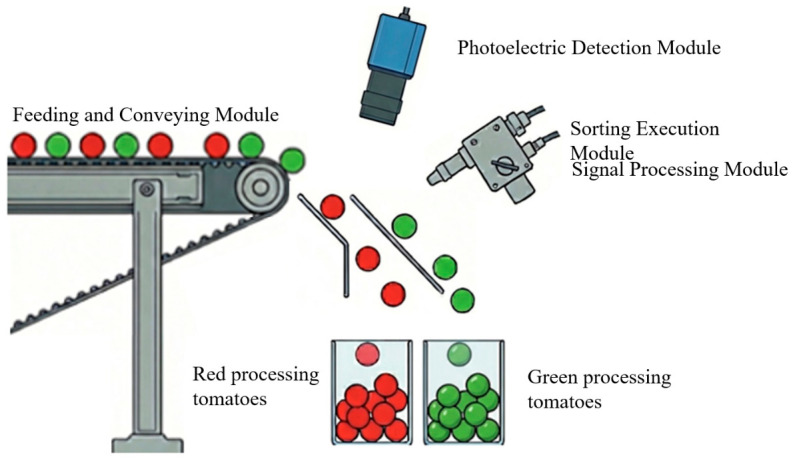
Schematic architecture of the intelligent color sorting system, illustrating the synergistic workflow of the four core modules: feeding and conveying, photoelectric detection, signal processing, and sorting execution.

The design logic of tomato intelligent color sorting equipment follows the closed-loop control theory of perception-to-decision-to-execution, as shown in Figure 5, and its engineering implementation is primarily accomplished through the collaboration of four core modules: feeding and conveying, photoelectric detection, signal processing, and sorting execution. During the operation process, the feeding system first utilizes a vibratory feeder in conjunction with a high-speed conveyor belt to rectify unstructured scattered raw materials into a single-layer and velocity-stable ordered material stream, thereby minimizing the interference of motion blur on subsequent imaging. Subsequently, the photoelectric detection unit captures high-fidelity optical image features of the material in real-time as it flows through the detection zone under a specific illumination environment. The signal processing system adopts a heterogeneous computing architecture to perform high-speed feature extraction and logical discrimination on massive data streams, precisely pinpointing the spatial coordinates of defective fruits or impurities. Finally, the sorting execution system actuates terminal pneumatic nozzles or mechanical fingers based on control instructions to alter the motion trajectory of the target material within a millisecond-level time window, thereby achieving efficient separation of acceptable products from impurities.

### 3.2. Feeding and Imaging Hardware

The architecture of the optical inspection system directly dictates the recognition accuracy and response speed of the color sorting equipment. As shown in Table 2, which compares different core detection components in color sorters, the industry currently widely adopts high-frequency LED cold light sources in the illumination subsystem to replace traditional halogen lamps. High-frequency LED light sources offer high spectral purity, low thermal radiation, and short response times. They provide a bright, uniform illumination field that minimizes shadows and stroboscopic effects during imaging. Additionally, their low heat output prevents tomato skin from scorching.

Regarding the selection of core detection components, a hierarchical configuration technical route has emerged to address the industrial characteristics of tomato color sorting operations, which involve multi-channel parallelism, extremely fast material flow rates, and massive throughput. For bulk rough sorting stages requiring extremely high processing capacity, the system relies primarily on high-speed color sensors based on photodiode arrays. Such sensors directly convert spectral intensity in specific wavebands into analog voltage signals or digital pulses without the need for complex image matrix reconstruction, thereby achieving microsecond-level ultra-high response speeds capable of precisely matching high-speed material flows of several meters per second. Conversely, in scenarios requiring the identification of minute lesions, shape defects, or high-precision grading, high-resolution color CCD or CMOS imaging sensors are indispensable, as their advantage in high spatial resolution compensates for the color sensors’ inability to perceive texture and morphology [88,89]. Furthermore, multispectral cameras have been introduced into the system specifically for the rejection of malignant impurities such as red soil clods, stones, and internal rot; by fusing the differences in spectral response between visible light and near-infrared wavebands, they effectively resolve the technical difficulty of distinguishing metameric impurities [90,91].

In the material conveying stage, the dynamic stability of the feeding device is a prerequisite for ensuring detection accuracy. Traditional chute-style feeding structures utilize gravitational acceleration to slide materials down a chute; although structurally simple, they are highly prone to inducing irregular rolling and bouncing for spherical or ellipsoidal processing tomatoes [92,93]. This uncontrolled state of motion causes random deviations in the position and pose of the fruit as it passes through the detection zone, severely reducing the hit rate of the actuator. In contrast, belt-type conveying devices demonstrate superior adaptability. This structure utilizes a high-speed, smoothly running horizontal conveyor belt to carry materials, in conjunction with V-grooves or flexible rectifying mechanisms, forcing the tomatoes to remain relatively static or move in a uniform straight line when entering the optical field of view. This design not only eliminates the influence of motion blur on imaging quality but also ensures the predictability of the material trajectory, providing a reliable physical foundation for achieving precise pinpoint rejection.

**Table 2:** Comparison of different core detection components in color sorters.

Comparison Dimension	Traditional Photoelectric Type	CCD/CMOS Imaging Type	Near-Infrared (NIR) Spectroscopy Type	X-Ray Transmission Type
Core Imaging Principle	Based on photodiodes detecting sudden changes in single-point reflection intensity; lacks image reconstruction capability.	Based on linear or area array image sensors to acquire RGB color space and spatial geometric texture information [94].	Based on the absorbance differences in organic molecules (O-H, C-H bonds) in specific infrared wavebands to perform spectral fingerprint analysis [95,96].	Based on the attenuation rate differences in X-rays penetrating objects, reflecting the atomic number and density distribution of the material.
Recognition Accuracy	Can only distinguish between light/dark and large color differences; lacks spatial resolution.	Pixel-level resolution reaches up to 0.05 mm, enabling the recognition of minute lesions.	High spectral resolution, but spatial resolution is typically lower than that of visible light cameras.	High-density resolution; capable of detecting metal or stones with a diameter greater than 1 mm.
Advantageous Detection Targets	Impurities with extreme color differences, such as large green stems/leaves and white woven bag fragments.	Green fruit, mold spots, cracked/leaking fruit, and irregularly shaped soil clods.	Internal rot, sugar content grading, and metameric impurities (same color, different spectrum).	Stones wrapped in slurry, metal particles, glass fragments, and high-density hard soil clods.
Main Technical Limitations	Unable to recognize impurities with similar shapes, textures, or colors; high false rejection rate.	Highly affected by ambient lighting; cannot penetrate fruit skin; difficult to distinguish red stones that are extremely similar in color to tomato skin.	Expensive sensors; sensitive to moisture; requires establishing specific spectral calibration models for different varieties.	High radiation safety protection requirements; core components like tubes have short lifespans and are expensive; unable to detect low-density impurities.
Processing Capability	Simple signal processing with microsecond-level response speed.	Relies on high-performance GPU/FPGA for real-time image processing.	Large hyperspectral data volume; real-time processing places extremely high demands on computing power.	Limited by detector readout speed and image reconstruction algorithms.
Best Applicable Process	Installed on harvesters or inlets for rapid rejection of large amounts of obvious stem/leaf impurities.	Mainstream factory model used for finished product grading and appearance quality control.	Used for raw material screening in the production of high value-added products.	A critical checkpoint on the production line designed to effectively reduce the presence of high-density foreign bodies, thereby significantly enhancing food safety.

### 3.3. Rejection Mechanism

**Figure 6:**
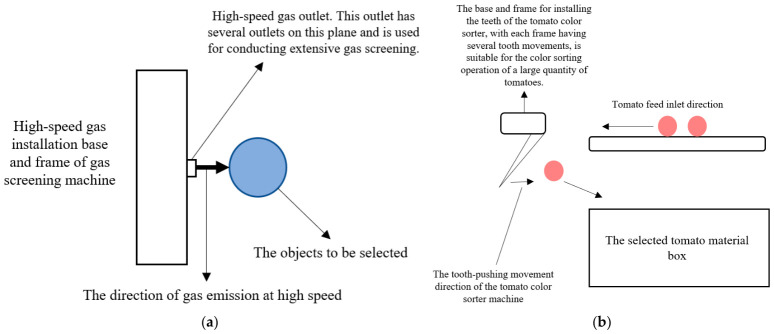
Typical actuation modes for material separation. (**a**) Non-contact pneumatic ejection; (**b**) contact-based rigid finger rejection.

As the terminal control unit of the color sorting system, the actuator’s dynamic response characteristics and mechanical behavior directly determine the sorting accuracy and raw material quality. Currently, two mainstream forms exist in industrial applications: pneumatic air jets and mechanical fingers. As shown in Figure 6a, the air-blowing mechanism is based on fluid mechanics principles, utilizing high-speed solenoid valves to release high-pressure airflow for the non-contact purging of foreign matter. Although this technology performs excellently in the sorting of lightweight materials such as grains, it reveals obvious dynamic limitations when dealing with large-mass processing tomatoes. Due to the large individual mass and significant inertia of mature tomatoes, the airflow struggles to provide sufficient impulse within a millisecond-level timeframe to effectively alter their motion vectors, often leading to rejection trajectories deviating from the predetermined path and severely constraining the sorting removal efficiency.

However, traditional continuous pneumatic rejection systems often struggle with the significant inertia limitations associated with large-mass fruits, such as industrial tomatoes. To address this bottleneck, recent fluid dynamics research has increasingly focused on high-pressure, pulsed air jet configurations. Building on insights into controlling disruptive soil airflow, the application of high-pressure pulsed air jets in sorting leverages instantaneous bursts of kinetic energy to precisely overcome the inertia of large-mass fruits, optimizing energy use while ensuring accurate ejection and minimal product damage [97]. Unlike continuous flows, pulsed air jets can deliver a concentrated, instantaneous burst of kinetic energy, effectively overcoming the inertia of heavier objects while optimizing compressed air consumption. Furthermore, these advanced aerodynamic configurations allow for precise spatial and temporal control over the ejection force, improving sorting accuracy for large-mass agricultural products without inducing excessive biomechanical damage.

In contrast, as shown in Figure 6b, the mechanical finger mechanism directly acts on the material through electromagnetically driven rigid fingers, capable of transferring greater kinetic energy, thus making it more suitable for the separation of bulky materials. However, traditional rigid finger designs overlook the vulnerability of biological materials. At the moment of high-speed collision, an approximate point contact state is formed between the rigid actuator and the viscoelastic tomato fruit, causing severe stress concentration in the contact area. This instantaneous high-intensity impact load easily exceeds the biological strength threshold of the fruit skin, inducing latent bruising or visible rupture. Consequently, the flexible design of actuators has become a key trend in technological evolution, specifically, by applying flexible composite materials or developing bionic flexible structures to increase the contact area and cushion impact energy, thereby minimizing mechanical damage while ensuring effective separation.

While each sensing modality offers unique advantages for tomato quality assessment, a critical synthesis reveals inherent trade-offs between detection depth and environmental robustness. Visible imaging provides the highest throughput and lowest cost, yet its efficacy is severely limited in open-field environments where dust and mud often mimic the spectral signatures of the fruit (metamerism). In contrast, NIR and X-ray technologies offer superior penetration, enabling the detection of internal defects and foreign objects (stones/clods) shielded by mud. However, the trade-off for this robustness is a significant increase in system complexity and cost. Specifically, X-ray systems introduce radiation safety constraints and bulky hardware requirements that currently hinder their seamless integration into compact, mobile harvester platforms. Thus, a multi-sensor fusion approach appears to be the most viable compromise for achieving high-precision grading under unstructured field conditions.

To systematically evaluate the feasibility of deploying these diverse sensing modalities on mobile harvesting platforms, a comparative framework is established (Table 3). This synthesis highlights the inherent trade-offs among external/internal defect detection capabilities, environmental robustness, and system performance metrics such as throughput and computational burden. As illustrated, while visible imaging provides the highest throughput and readiness for harvester-mounted deployment, its vulnerability to dust and inability to detect internal defects necessitate complimentary technologies. Conversely, modalities like X-ray and tactile sensing offer superior internal inspection and robustness but currently face significant bottlenecks in physical integration and high-speed operations.

## 4. Visual Recognition and Algorithm Models

Traditional machine vision sorting technology has long relied on handcrafted feature extraction operators and deterministic logic rules; its core objective is to separate target fruits from complex backgrounds and determine their quality through pixel-level color analysis and morphological operations. In the basic color feature processing stage, algorithms typically first convert the acquired raw images from the device-dependent RGB color model to the Hue–Saturation–Value (HSV) or CIE Lab color space defined by the International Commission on Illumination. Since the three-channel values of the RGB model exhibit extremely high sensitivity to changes in external light intensity, making it difficult to adapt to dynamically changing lighting conditions in unstructured field environments, the HSV space allows for the orthogonal separation of color and brightness information, making threshold segmentation based on the hue component, the most universal approach for distinguishing mature red fruits from green stems/leaves, immature green fruits, and backgrounds. After determining the Region of Interest (ROI), adaptive thresholding algorithms such as the maximum between-class variance method (Otsu’s method) are widely used for image binarization to achieve the preliminary separation of foreground and background.

**Figure 7:**
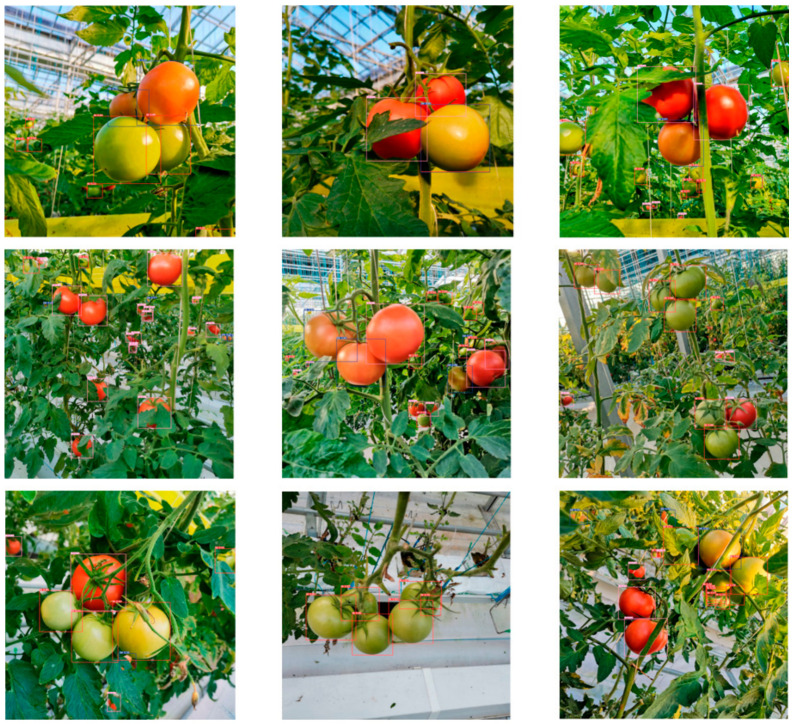
The red box represents the location of the tomato target [100].

Building upon the completion of pixel-level segmentation, feature extraction based on geometric morphology and texture details becomes a critical step for the further rejection of foreign matter [98,99,100,101], as shown in Figure 7. Targeting the biological characteristic that processing tomato fruits mostly appear as regular ellipses, algorithms construct shape descriptors by calculating geometric parameters of connected regions—such as circularity, rectangularity, eccentricity, and major axis aspect ratio—thereby effectively identifying and eliminating irregularly shaped soil clods, stones, and broken plant residues. Meanwhile, second-order statistical texture features extracted using the Gray-Level Co-occurrence Matrix (GLCM), such as energy, entropy, contrast, and correlation, are used to quantify the smoothness and gray-level distribution patterns of the fruit surface, thus distinguishing to a certain extent between healthy fruits with smooth skins and diseased samples exhibiting shriveling, cracking, or mold mycelium.

In early research, methods based on traditional machine vision and handcrafted feature extraction were widely applied. Prabha et al. [102] extracted the average color intensity and morphological features from image histograms for banana maturity detection, verifying that color intensity is a significant feature for distinguishing maturity. Dairath et al. [103] developed a robotic harvesting and grading system, which utilized HSV color space mask processing to calculate the proportion of fruit defects, achieving rapid grading. Focusing on hawthorns, Azadnia et al. [104] achieved a classification accuracy of 99.16% by extracting geometric, color, and GLCM texture features combined with Quadratic Discriminant Analysis (QDA) and Artificial Neural Networks (ANNs). Ropelewska [105] achieved high-precision differentiation of apple varieties by extracting multi-channel texture features of the fruit skin and cross-sections combined with Bayesian networks.

In recent years, Convolutional Neural Networks have occupied a dominant position in fruit detection within complex scenes by virtue of their powerful capability for autonomous feature extraction. Targeting issues regarding occlusion and illumination in natural environments, numerous scholars have implemented improvements to the YOLO series algorithms. Li [100] proposed the MHSA-YOLOv8 model, which introduces a Multi-Head Self-Attention (MHSA) mechanism to enhance feature extraction, effectively improving the accuracy of tomato maturity grading. To further improve lightweight performance and robustness for multi-span greenhouse environments, Wang [106] utilized Cycle GAN to perform adaptive restoration on images of occluded fruits.

Although two-dimensional image detection has developed rapidly, its data consists of massive pixels containing a large amount of redundant background information, imposing high demands on hardware computing power. In contrast, one-dimensional signal analysis based on spectroscopy or sensors possesses the advantages of small data volume and fast processing speed. In the field of spectral analysis, Saha [107] utilized a dual-wavelength LiDAR sensor to acquire point cloud return intensity signals of tomatoes and calculated the LiDAR-NDVI index, successfully establishing a prediction model for tomato chlorophyll content and maturity, thereby demonstrating the feasibility of inverting internal quality from photoelectric signals. To mine the deep features of one-dimensional signals, One-Dimensional Convolutional Neural Networks (1D-CNN) have been widely applied in processing various time-series and spectral signals, providing important methodological support for this study.

Although traditional image processing algorithms have demonstrated a certain degree of usability in ideal controlled environments with constant lighting and dispersed materials, the limitations they expose during actual industrial high-throughput operations are becoming increasingly prominent. First, threshold segmentation methods based on fixed or shallow statistical rules lack robustness against environmental changes; they are highly prone to under-segmentation or over-segmentation when facing instantaneous fluctuations in natural light or shadow occlusion [106,108,109]. Secondly, handcrafted feature extractors possess weak generalization capabilities and struggle to exhaustively cover the complex and variable forms of field impurities; relying solely on shallow features like color and shape often fails to achieve precise classification—particularly when facing red soil clods that are extremely similar in color to mature tomatoes, or stems and leaves whose spectral characteristics highly overlap with green fruit—leading to a significant rise in the false selection rate. Furthermore, traditional algorithms appear inadequate when dealing with fruit overlap and adhesion problems; simple watershed algorithms or morphological opening and closing operations often struggle to accurately segment densely stacked material flows, severely limiting the detection accuracy and operational efficiency of sorting equipment in high-speed, high-yield scenarios.

The transition from traditional image processing to CNN-based vision has drastically improved the robustness of grading systems against occlusion and variable lighting. However, this analytical power introduces a critical trade-off in terms of computing demand and system cost. High-performance deep learning models require specialized edge-computing hardware (e.g., GPUs or FPGAs) to maintain real-time throughput during high-speed harvesting. It is critical to distinguish here that while complex, heavy-weight deep learning models show exceptional accuracy in stationary or laboratory setups (promising but not yet practical), the severe thermal loads and limited embedded computing capacities on a mobile harvester restrict field-ready applications strictly to ultra-lightweight, optimized architectures. In the harsh environment of open-field production—characterized by high temperatures and mechanical vibrations—this reliance on intensive computing hardware poses significant challenges for thermal management and system reliability. Therefore, future research must balance the pursuit of marginal gains in detection accuracy with the need for lightweight, energy-efficient architectures that can be deployed on cost-effective embedded systems. Although deep learning models have shown remarkable success in harvesting apples and papayas under structured orchard lighting, direct empirical evidence for processing tomatoes in unstructured, high-dust, and high-vibration open-field environments remains scarce and requires distinct algorithmic lightweighting.

## 5. Low-Damage Sorting and Actuator Optimization

### 5.1. Analysis of Mechanical Damage Mechanisms

**Figure 8:**
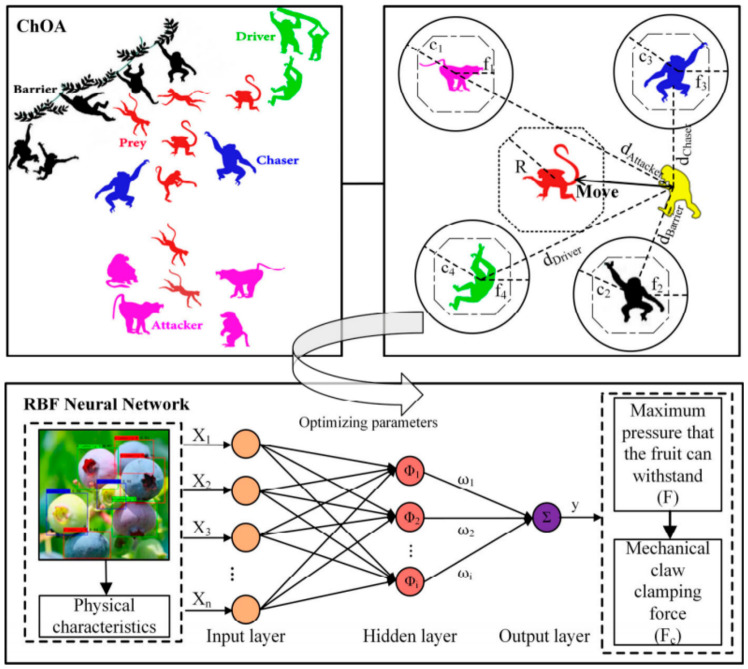
Using the ChOA algorithm to optimize the RBF prediction model for fruit hardness prediction and adjusting the gripper force [143].

As a type of biological soft matter possessing complex rheological properties, the fruit structure of the processing tomato consists of the exocarp, mesocarp parenchyma tissue, and internal gelatinous placenta. Throughout the entire color sorting workflow, tomato fruits inevitably undergo contact coupling with mechanical components [110,111,112,113,114,115,116]; their modes of damage primarily depend on the loading rate and the nature of the load, typically classified into two major categories—quasi-static compression damage and dynamic impact damage—with essential differences existing between their failure mechanisms [117,118,119,120,121,122]. Specifically, while quasi-static compression typically manifests as continuous internal tissue yielding under prolonged stress, dynamic impact tends to trigger instantaneous epidermal rupture and irreversible cell wall fracture [123,124,125,126]. Li et al. [127] established a 3D multi-scale finite element model of a tomato comprising the cuticle, pericarp framework, and fluid cavity by introducing microscopic cell units; they discovered that the damage volume caused by vertical compression follows an S-shaped model relative to the deformation rate, revealing that microscopic cell yielding often initiates within the interior of the pericarp. While the aforementioned study provides direct microstructural evidence for tomatoes, the finite element modeling of biomechanical damage in processing tomatoes is still in its developing stage. To build robust predictive models, researchers frequently draw upon indirect methodological evidence transferred from other well-studied horticultural crops. For instance, regarding mechanical grasping simulation, Ji et al. [128] developed a viscoelastic finite element composite parallel model for apples based on the Burgers model; they simulated stress variations under constant-velocity grasping, uncovering the mechanism whereby the skin sustains damage first due to plastic deformation. Aina et al. [129] modelled papaya mechanical behavior under compression using finite element analysis, concluding that the viscoelastic model yielded the highest accuracy while loading direction significantly affected stress distribution. Chen et al. [130] investigated apple compression damage characteristics using multi-scale finite element analysis and mechanical tests, concluding that internal stress positively correlates with compression displacement but negatively with storage duration, validating the model’s high accuracy. Chen et al. [131] quantified collision-induced apple damage using explicit dynamic finite element analysis and response surface methodology, concluding that impact height significantly affects damage severity while the developed models accurately predict bruising characteristics. Chen et al. [132] investigated ‘Red Fuji’ apple damage patterns using explicit dynamic finite element simulations, concluding that impact height is the primary damage factor and validating a regression model for accurate susceptibility prediction. Although these cross-crop studies provide valuable theoretical frameworks and finite element modeling methodologies, it is crucial to acknowledge the limits of transferring these findings directly to processing tomatoes. Biomechanically, processing tomatoes possess a highly specific multiphase composite structure characterized by a thin exocarp, dense mesocarp parenchyma, and high-moisture gelatinous locular cavities. This differs significantly from the relatively uniform, firm, and lower-moisture fleshy parenchyma of apples, pears, or papayas. Consequently, the viscoelastic stress propagation and cell-rupture thresholds are fundamentally different. Furthermore, industrial handling conditions for processing tomatoes—which involve aggressive multi-ton combine harvesting, severe operational vibrations, and bulk unloading—present a much harsher dynamic environment compared to the individual, careful handling typical of fresh-market fruit grading. Therefore, while cross-crop finite element models offer foundational insights, their rheological parameters and failure criteria must be rigorously recalibrated using species-specific empirical data before being applied to the damage-mechanics analysis of processing tomatoes. Ding et al. [133] addressed inaccurate damage prediction in Korla pears via a micro-CT-based FDEM model, concluding that a two-stage parameter approach significantly enhances accuracy by capturing abrupt mechanical transitions across maturity stages. Gao et al. [134] addressed fruit mechanical injury via a systematic review of numerical simulation techniques, concluding that methods like FEM and DEM provide critical theoretical support for optimizing post-harvest handling and reducing quality losses. Hao et al. [135] addressed harvest-induced compressive damage in Cerasus humilis via mechanical testing and finite element simulation, concluding that the fruit exhibits anisotropic properties with higher axial strength, and the model accurately predicts fracture initiation and propagation. Li et al. [136] addressed the quantitative characterization of apple impact damage via an elastic modulus-based prediction model using hyperspectral imaging, concluding that the spectrally corrected model significantly improves prediction accuracy by eliminating fruit size effects. Li et al. [137] addressed apple harvesting damage and inefficiency via an optimized airflow-cushioned conveyor, concluding that optimal operational parameters significantly reduced mechanical injury while more than doubling harvesting speed compared to manual methods. Marquardt et al. [138] addressed fruit damage potential in cross-section constrictions via HLBM-DEM simulations, concluding that elevated solid volume fractions and constriction ratios significantly increase mechanical stress and subsequent injury risks. Urbańska et al. [139] addressed the determinants of kiwifruit mechanical susceptibility via compression testing, concluding that Young’s modulus declines primarily due to physiological softening, while water loss and compression velocity exert negligible influence on stiffness. Wang et al. [140] addressed the compressive mechanical properties of Rosa sterilis fruit via finite element analysis and experimental verification, concluding that the fruit exhibits significant directional sensitivity which the simulation accurately predicts. Wei et al. [141] addressed rheological deformation during clustered fruit harvesting via a dynamic model combining viscoelasticity and multibody contact, concluding that the approach accurately quantifies mechanical damage thresholds and stress characteristics. Xia et al. [142] addressed internal quality deterioration in Korla pears under multi-type damage via physiological monitoring and kinetic modeling, concluding that varying damage modes distinctively accelerate decay, accurately predicted by the constructed quality evolution models. Yin et al. [143] addressed blueberry gripping damage via a ChOA-RBF neural network based on visual features, as shown in Figure 8, concluding that the model accurately predicts hardness to enable adaptive force control and minimize mechanical injury. Zeeshan et al. [144] addressed damage rates in robotic orange harvesting via experimental testing and regression modeling, concluding that perpendicular gripping, minimal occlusion, and optimal illumination are critical for minimizing fruit injury. Zhou et al. [145] addressed post-harvest carrot mechanical damage via a multi-scale viscoelastic–plastic-bonded particle model, concluding that the integrated approach accurately predicts deformation forces and visualizes internal crack propagation under varying loading conditions.

Quasi-static compression damage mainly occurs during the material conveying, rectifying, and stacking stages [146,147,148,149]. In this process, tomatoes are subjected to continuous compressive forces from conveyor belts, guide plates, or adjacent fruits, with a relatively low loading rate. According to rheological models of biological materials, fruits exhibit creep and stress relaxation phenomena under low-speed loading. When external compressive stress exceeds the bio-yield limit of the fruit tissue, although the skin may maintain macroscopic integrity due to high cellulose strength, the structural stability of internal parenchyma cells is compromised. At this point, microscopic fractures occur in cell walls, and cell membrane permeability changes, leading to cell sap leakage and the loss of turgor pressure. This type of damage is often difficult to detect visually immediately and is termed as latent bruising; however, during subsequent storage and transportation, the contact between polyphenol oxidase and substrates within the damaged tissue triggers enzymatic browning, seriously affecting the color and flavor of the processing finished product.

In contrast, dynamic impact damage is the primary failure mode in the color sorting rejection stage, with its occurrence typically completing within a millisecond-level time window. When pneumatic nozzles or mechanical fingers strike the target fruit at extremely high speeds, or when the fruit impacts the receiving hopper wall at high speed after rejection, massive kinetic energy is instantly converted into the deformation potential energy of the fruit. Since processing tomatoes are viscoelastic bodies with high water content, they possess extremely high sensitivity to strain rates. Under high-speed impact, internal fluids cannot dissipate energy through flow in time, causing local tissue stiffness to exhibit dynamic hardening characteristics. Once the shear stress generated by the transient shock wave exceeds the binding strength between the skin and flesh or the ultimate tensile strength of the skin itself, the fruit surface undergoes visible rupture, resulting in juice loss and deeper tissue ulceration.

To quantitatively analyze the aforementioned damage processes, the academic community has widely introduced the Hertzian contact theory as a core analytical tool [150,151,152,153,154]. This theoretical model simplifies the collision between the rejection mechanism and the tomato into a contact problem between a rigid plane or curved surface and an elastic sphere [155]. According to Hertzian contact mechanics formulas, when a rigid mechanical finger contacts a flexible tomato, an approximate point contact state is formed due to the extremely small contact area [156]. In the center of the contact region, the maximum contact pressure is directly proportional to the cube root of the external load and inversely proportional to the two-thirds power of the equivalent radius of curvature of the contact area. This implies that when using traditional rigid fingers with small radii of curvature for rejection, even if the applied striking force is not huge, extremely high stress concentration phenomena will occur at the minute contact point [141,157,158]. It is crucial to acknowledge that a significant portion of the foundational Hertzian contact models and multi-scale damage theories discussed herein—such as drop-impact bruising models—were initially developed for apples, pears, and papayas. Applying these indirect models to processing tomatoes poses inherent limitations, as processing tomatoes exhibit much higher water content, thinner pericarps, and are subjected to far harsher multi-impact collisions inside a combine harvester.

This highly concentrated contact stress forms a semi-ellipsoidal stress field distribution inside the fruit, where the maximum shear stress typically appears at a specific depth below the contact surface. When the peak shear stress at this location breaches the shear strength threshold of the tomato parenchyma tissue, the subcutaneous tissue yields and flows plastically first, forming so-called subcutaneous bruising. For processing tomatoes, which have thin skins and abundant internal juice, their ability to resist Hertzian contact stress is weaker than that of common fresh-market tomatoes; therefore, they are more prone to internal-to-external tissue disintegration when subjected to mechanical strikes of equivalent intensity. In summary, the key to reducing mechanical damage lies in optimizing contact geometric parameters to increase the contact area, thereby lowering the peak Hertzian contact pressure, and controlling the impact kinetic energy to maintain it within the damage threshold of biological tissues.

### 5.2. Design of Flexible Actuators

Addressing the intrinsic defects of traditional rigid actuators that easily cause mechanical damage during tomato sorting, the introduction of soft robotics technology and flexible material design has emerged as a core pathway for actuator optimization. Unlike rigid linkage mechanisms that rely on kinematic pairs for deterministic motion transmission, soft robotics technology utilizes the nonlinear large deformation properties of the material itself to adapt to unstructured environments, possessing natural compatibility with the biomechanical characteristics of processing tomatoes—thin skins, thick flesh, and susceptibility to damage. By constructing end-effectors with passive compliance, it is possible to physically achieve the buffering and dissipation of collision energy, thereby fundamentally solving the challenge of momentum-induced damage during high-speed rejection operations [159].

In the dimension of material selection, silicone rubber and Thermoplastic Polyurethane (TPU) elastomers, which possess high viscoelasticity and excellent wear resistance, are the preferred base materials for constructing flexible end-effectors. These polymer materials not only possess biocompatibility, meeting food contact safety standards, but also, more importantly, endow the actuator with intrinsic compliance at the instant of contact due to their extremely low Young’s modulus characteristics [160]. When a flexible actuator collides with the tomato surface at high speed, the elastic deformation of the material itself can effectively absorb and store part of the impact kinetic energy, converting it into internal elastic potential energy, thereby exerting a significant damping and buffering effect. Furthermore, the excellent wear resistance and fatigue resistance of polyurethane materials ensure that the actuating components maintain a stable mechanical response even under millions of high-frequency reciprocating strikes, adapting to the harsh continuous operating conditions of color sorters.

Structural innovations based on soft robotics have further enhanced low-damage sorting performance, wherein bio-inspired design concepts are widely applied in the configuration optimization of rejection components [161,162,163]. The Fin Ray Effect structure, inspired by the movement mechanism of fish fins, is currently a research hotspot. This structure typically consists of a triangular mesh formed by two flexible ribs and intermediate elastic crossbeams; when subjected to external pressure on one side, the entire structure actively bends towards the direction of the force. When applied to the finger design of color sorters, at the moment of contact with the tomato, the finger is no longer a simple impactor but generates an adaptive wrapping action based on the fruit’s outer contour. This compliant deformation behavior transforms the originally concentrated point contact load into a uniformly distributed surface contact load, greatly expanding the area of force application.

**Figure 9:**
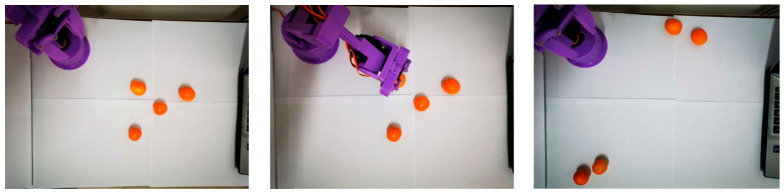
Tomato target positioning test [165].

Analyzing deeply from the perspective of contact mechanics, the introduction of flexible structures essentially reconstructs the momentum transfer mechanism of the collision process, as shown in Figure 9 [164,165]. The hysteretic deformation of flexible materials significantly prolongs the physical contact time of the collision; according to the momentum theorem, under the boundary condition of transmitting the same rejection impulse, the prolongation of action time directly leads to a substantial attenuation of the peak instantaneous impact force. Simultaneously, the expansion of the contact interface causes the contact pressure to drop exponentially, thereby ensuring that the maximum shear stress in the contact region remains consistently below the biological yield threshold of the tomato subcutaneous tissue. Targeting the high-throughput operational characteristics of color sorters, current research focuses mainly on pneumatic flexible fingers and rigid–flexible coupled dials. By wrapping multi-chamber airbags around a rigid skeleton or designing hollow fluid-driven cavities, it is possible to ensure sufficient rejection driving force to alter the trajectory of large-mass tomatoes while utilizing the flexible deformation at the tip to achieve non-destructive contact, achieving a dynamic balance between high-speed sorting and low damage rates.

### 5.3. Simulation and Parameter Optimization

In the research and development of intelligent tomato sorting equipment, relying solely on the trial manufacture and testing of physical prototypes often presents limitations such as long cycles, high costs, and the difficulty of observing millisecond-level transient contact processes. With the maturity of Computer-Aided Engineering technology, utilizing numerical simulation methods to construct virtual prototypes and conduct Multiphysics simulations has become a core research paradigm for deeply revealing sorting mechanisms, optimizing mechanism designs, and establishing optimal operating parameters. Current research mainly focuses on co-simulation using the multibody dynamics software ADAMS and the finite element analysis software ANSYS with its explicit dynamic module LS-DYNA, aiming to seek optimal solutions from the two dimensions of kinematic stability and dynamic low-damage characteristics [166,167]. While finite element crash simulations have been extensively validated on apples and peaches (indirect evidence), researchers are now beginning to build direct evidence for processing tomatoes by developing multi-layered models that distinguish between the exocarp, mesocarp parenchyma, and internal gelatinous placenta. However, relying solely on kinematic simulation is insufficient for quantifying microscopic damage mechanisms within the fruit at the moment of impact. Consequently, explicit dynamic finite element analysis, utilizing platforms such as ANSYS/LS-DYNA, has emerged as a pivotal tool for investigating the damage thresholds of biological materials. A critical challenge in this domain is the construction of high-fidelity biomechanical models. While existing studies often simplify the tomato into a single homogeneous viscoelastic sphere, this approach frequently fails to capture complex internal stress transmission patterns. Conversely, advanced research prioritizes the development of multi-layered, refined models that distinguish between the exocarp, mesocarp parenchyma, and the internal gelatinous placenta. These sophisticated models enable not only the visualization of Von Mises stress distributions in the contact zone but also the elucidation of shear stress propagation paths within subcutaneous tissues. This micromechanical perspective provides a theoretical basis for actuator optimization. By correlating peak contact pressures with varying curvature radii, elastic moduli, and impact velocities, a ‘safe operation window’—defined by the bio-yield limit—can be precisely delineated. This signifies a paradigm shift in sorting equipment design, moving from empirical ‘trial-and-error’ approaches to ‘predictive design’ strategies based on digital twins.

Multibody dynamics simulation primarily focuses on resolving motion trajectory control issues during material conveying and rejection processes. Researchers utilize ADAMS software to construct parameterized virtual prototypes containing conveyor belts, tomato particles, and rejection actuators, enabling precise simulation of the projection, rolling, and collision trajectories of tomatoes under high-speed motion states [168,169]. By applying boundary conditions such as gravity, friction, and aerodynamic drag, the simulation model can quantitatively analyze the effects of different belt speeds, rejection timings, and impact angles on the material separation success rate. Particularly, in research targeting non-spherical processing tomatoes, multibody dynamics simulation is used to verify the effectiveness of belt-type rectifying mechanisms; specifically, by analyzing the centroid displacement and attitude angle changes in the fruit during conveying, the geometric parameters of the rectifying device are optimized to ensure tomatoes enter the rejection zone in the most stable long-axis orientation, thereby maximizing the reduction in false rejection risks caused by random tumbling [170].

However, kinematic simulation alone cannot quantify the degree of mechanical damage to the fruit at the instant of collision. Therefore, explicit dynamic finite element analysis based on ANSYS or LS-DYNA has become a key means for exploring microscopic damage mechanisms [171,172]. In this field, constructing high-fidelity biomechanical models of tomatoes acts as the foundation for simulation. Researchers typically use experimental data on tomato rheological properties, employing viscoelastic or hyperplastic constitutive models to define the material properties of the flesh and skin, and establish multi-layer refined finite element meshes comprising the epidermis, pericarp, and gelatinous placenta. Utilizing LS-DYNA’s powerful transient nonlinear solution capabilities, it is possible to simulate the entire process of rigid or flexible fingers impacting the tomato surface at different velocities, intuitively visualizing the stress wave propagation laws, strain energy density distribution, and equivalent stress contours in the collision contact area. Simulation results indicate that when the peak contact stress exceeds the bio-yield limit of the tomato tissue, the element mesh fails, corresponding to skin rupture or subcutaneous bruising in the physical world [173,174]. By comparing the stress responses of actuators with different shapes and elastic moduli materials under collision, the significant advantages of flexible end-effectors in reducing Hertzian contact stress and protecting fruit integrity can be verified from a theoretical perspective [175,176].

Based on the aforementioned simulation data, parameter optimization design becomes the bridge connecting theoretical analysis and engineering application. Research typically employs Orthogonal Experimental Design or response surface methodology (RSM), setting structural parameters of the rejection mechanism (such as finger length, radius of curvature, coating thickness) and operational parameters (such as conveyor speed, impact pressure, delay time) as design variables, while defining sorting hit rate and fruit damage rate as multi-objective optimization functions [177,178]. By conducting large-sample virtual iterative experiments on a co-simulation platform, mathematical regression models between design variables and response objectives can be constructed, thereby solving for the optimal parameter combination that satisfies the balance point between low loss and high efficiency. This digital twin-based forward design method not only significantly shortens the transition cycle from conceptual design to engineering prototype but also provides solid theoretical grounds and data support for the development of specialized color sorting equipment tailored to the thin-skinned and fragile characteristics of processing tomatoes.

With the development of computer simulation technology, the Discrete Element Method and Computational Fluid Dynamics are widely applied in the parameter optimization of sorting equipment [179,180,181], which is of great significance for reducing trial production costs and exploring the interaction mechanisms between materials and machinery. For lightweight, small fruit materials, pneumatic sorting offers the advantages of non-contact operation and minimal damage [182,183]. Compared to traditional physical sorting, intelligent sorting based on machine vision and sensor technology can more precisely reject foreign bodies and defective fruits.

Addressing the blind spot issue in single-view detection, Quan et al. [162] designed an online omnidirectional detection and sorting system for soybean seeds, achieving 360-degree blind-spot-free imaging of the seed surface through a special optical layout. Additionally, Zhou [165] designed and tested a general-purpose machine vision sorting device, verifying the real-time coordination between the visual recognition module and the actuator [184,185,186,187].

The implementation of compliant actuation and soft robotic effectors addresses the fundamental challenge of biomechanical damage control by providing a ‘damage-free’ contact interface for delicate tomatoes. Nevertheless, the critical trade-off here lies between protection and productivity (throughput). Viscoelastic materials typically exhibit slower dynamic response times and lower cycle frequencies compared to rigid mechanical or pneumatic systems, which may bottleneck the overall sorting speed. Furthermore, the durability of soft materials in the presence of abrasive field debris (vines and grit) remains a primary concern for long-term field deployment. For industrial-scale tomato harvesting, the optimal solution requires a hybrid design that synergizes the speed of rigid mechanisms with the cushioning properties of compliant interfaces, ensuring both high throughput and minimal post-harvest losses.

Pure visual detection struggles to fully assess the internal quality or firmness of fruits; thus, the fusion of multi-modal sensors has become a new trend for resolving flexible non-destructive testing. Addressing the issue that bulbous crops are prone to mechanical damage during high-speed sorting, the optimization of conveying and grading mechanisms is a current research hotspot. In terms of structural lightweighting and dynamic performance optimization of sorting equipment, the application of modern design methods is becoming increasingly widespread.

## 6. Challenges, Future Directions, and System-Level Integration of Electro-Mechanical Harvester-Mounted Sorting Technologies

### 6.1. Multi-Modal Information Fusion

With the increasingly stringent requirements for raw material quality in the processing tomato industry, the traditional detection mode relying solely on visible light machine vision has gradually reached its technological ceiling. When facing high-order sorting challenges such as the inclusion of complex impurities brought by field mechanized harvesting and internal physiological lesions of fruits, relying solely on apparent color and texture features often impedes precise rejection. Therefore, constructing a multi-modal information fusion perception system integrating visible light, near-infrared spectroscopy, X-ray transmission, and tactile sensing has become an inevitable trend to break through existing technical bottlenecks and achieve full-dimensional detection of internal and external quality.

**Figure 10:**
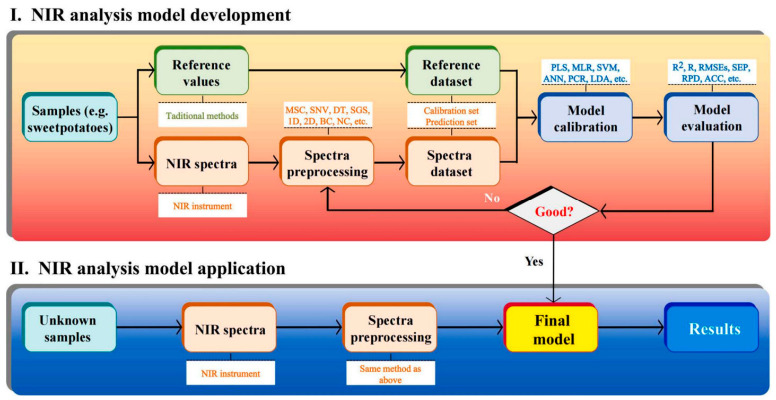
Flow chart of NIR analysis procedures [189].

As a foundational sensing method, visible light imaging primarily undertakes the tasks of fruit surface color grading and the recognition of explicit defects. However, its physical limitation lies in the inability to penetrate the peel to detect the state of internal tissues. To compensate for this deficiency, near-infrared spectroscopy technology has been introduced into the system to acquire the internal chemical fingerprints of materials, as shown in Figure 10 [188,189,190]. Based on the principle of specific absorption of near-infrared light by hydrogen-containing groups of organic molecules, near-infrared sensors can non-destructively invert the internal soluble solids content, acidity, and moisture distribution of tomatoes [191,192]. More critically, for internal browning, moldy core, and hollow fruits unrecognizable to the naked eye, NIRS can sensitively capture them through shifts in spectral absorption peaks and changes in scattering characteristics, thereby achieving perspective detection of internal quality without compromising fruit integrity [193,194,195].

**Figure 11:**
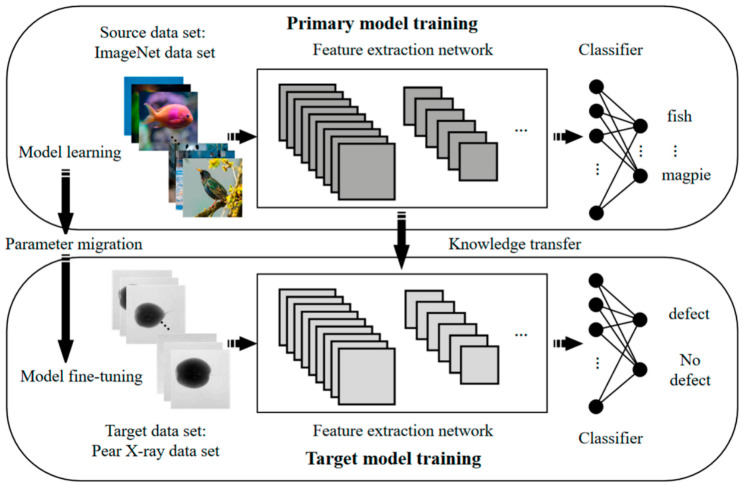
Schematic diagram of transfer learning process. The arrow represents the transfer of parameters and knowledge [201].

Pure spectral analysis still carries the risk of metameric misjudgment in targeting malignant impurities mixed in during mechanized harvesting—especially red soil clods and red stones extremely similar in color to mature tomatoes as well as metal foreign bodies wrapped in pulp. At this juncture, the intervention of X-ray transmission technology provides the system with a third-dimensional criterion based on density differences. As a type of high-energy electromagnetic wave, the penetrating ability of X-rays is negatively correlated with the atomic number and density of the material [196,197]. Utilizing X-ray imaging technology, the system can clearly present the internal density distribution image of the material [198,199]. Since the density of stones, glass, and metals is significantly higher than tomato biological tissue, they appear as dark regions in X-ray images, thereby enabling precise locking and rejection of these malignant hard impurities that could damage backend processing equipment from the massive material flow, ensuring the safety of the production line, as shown in Figure 11 [200,201].

Furthermore, regarding the soft rot and overripe softening issues unique to processing tomatoes, traditional non-contact optical detection often struggles to identify soft-injured fruits with intact skins but liquefied interiors. The rise of tactile sensing technology offers a brand-new physical perspective for solving this problem. By integrating piezoelectric or capacitive electronic skins at the flexible actuator end or specific contact-type detection stations, the system can acquire contact force feedback and deformation information within the millisecond-level instant of contact with the fruit. Based on viscoelastic mechanical models, tactile sensors can quantify the fruit’s stiffness modulus and restitution coefficient, thereby accurately identifying deteriorated fruits that appear red but are soft to the touch.

It is crucial to distinguish between mature applied technologies and emerging paradigms. While deep learning-based spatial coordinate regression and target detection have become standard practices in actual greenhouses, the fully ‘Embodied AI’ paradigm—where robots exhibit zero-shot generalization through large visual-language models—remains largely a future engineering vision. Bridging the gap between current simulation-based multi-modal interactions and robust, cost-effective field deployment is required. In summary, multi-modal information fusion technology is not a simple superposition of individual technologies but a deep integration based on the theory of information complementarity. At the data processing level, through heterogeneous data registration and feature-level fusion algorithms, the system maps the color/texture features of visible light, chemical composition features of NIRS, density features of X-rays, and rheological mechanical features of tactile sensing into a unified high-dimensional feature space. This comprehensive, multi-dimensional perception system eliminates the detection blind spots of single sensors, greatly enhancing system robustness in unstructured environments, and providing solid technical support for achieving the leap of processing tomatoes from surface sorting to high-quality intelligent grading that considers both internal and external attributes.

### 6.2. Multi-Modal Information Fusion for Tomato Color Sorting

While deploying advanced technologies such as X-ray transmission and heterogeneous computing on mobile combine harvesters offers theoretical advantages for high-density impurity sorting and real-time data processing, their practical implementation faces significant feasibility barriers that must be critically acknowledged.

Regarding X-ray systems, massive regulatory and safety constraints pose a primary challenge. Operating X-ray tubes require robust radiation shielding, which significantly increases the overall weight of the machinery, leading to altered centers of gravity, higher energy consumption, and increased soil compaction. Furthermore, the intense, high-frequency vibrations from the harvester engine and uneven field terrain severely threaten the durability of delicate vacuum tubes and high-voltage components. Additionally, continuous X-ray generation demands a highly stable and substantial power supply, which is difficult to maintain on conventional diesel-driven agricultural platforms. Economically, the exorbitant initial capital costs and specialized maintenance required for X-ray systems drastically reduce their viability for standard farm operations.

Similarly, deploying high-performance heterogeneous computing platforms directly in the field involves strict power limitations and extreme environmental challenges, such as severe dust, thermal management issues under direct sunlight, and moisture. Therefore, before these technologies can be broadly commercialized in mobile units, future research must focus on developing low-power, vibration-resistant solid-state X-ray sources and ruggedized, edge-computing hardware, or confine such advanced multi-modal sorting primarily to stationary, factory-level processing facilities.

**Figure 12:**
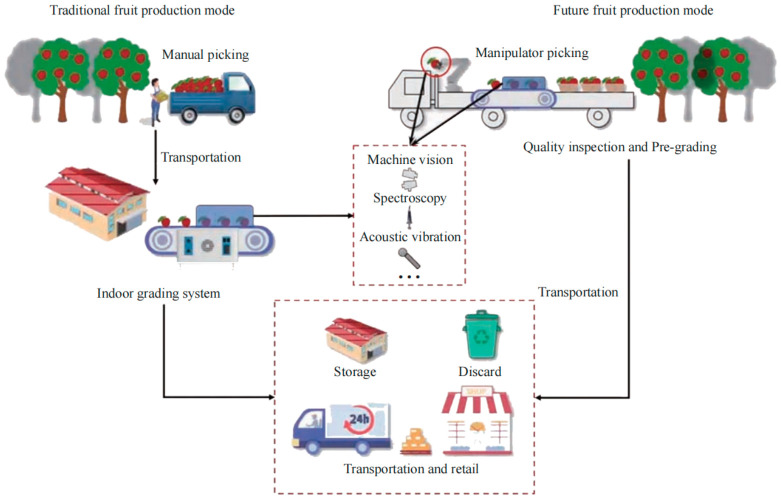
Labor-intensive traditional fruit production mode and future fruit production mode integrating infield picking, quality inspection and grading [205].

Field in situ sorting technology represents a potential advanced direction for future integration of modern agricultural equipment towards intelligence and integration; its core philosophy lies in breaking the traditional segmented operation mode of “harvest first, transport later, then sort” by directly embedding high-precision photoelectric sorting modules into large-scale combine harvesters, achieving spatial fusion and temporal synchronization of harvesting and sorting processes, as shown in Figure 12 [202,203,204,205,206,207,208]. For the vast processing tomato growing regions in Xinjiang, this operational mode holds significant economic and ecological value. It not only blocks invalid payloads like clods, stones, and stems/leaves from entering the logistics chain at the source, significantly reducing transportation costs and carbon emissions, but also timely removes moldy and damaged fruits to prevent cross-contamination by pathogenic microorganisms during long-distance transport, thereby maximizing the preservation of raw material freshness and processing quality.

The primary challenge in realizing this technological vision lies in constructing highly vibration-resistant imaging and actuation systems. When a combine harvester travels on unstructured field surfaces, subjected to terrain undulations and the coupling effects of rotating components like engines and threshing cylinders, the entire machine undergoes intense vibration across a wide frequency domain [209,210]. This complex vibration environment causes high-frequency jitter in the focal plane of the optical imaging system, inducing image motion blur and geometric distortion, which severely weakens the feature extraction capability of recognition algorithms [211]. To this end, research focuses on the design of multi-stage active and passive vibration damping mechanisms. In terms of passive damping, nonlinear vibration isolation mounts combining wire rope isolators and damping rubber are adopted to isolate low-frequency, large-amplitude road shocks [212]. Simultaneously, for the actuating mechanism, it is necessary to develop mechanical structures with self-locking functions to prevent accidental actions caused by machine jolts, ensuring the accuracy of rejection command execution.

Secondly, the establishment of an embedded edge-computing architecture is a prerequisite for achieving real-time in situ sorting. In contrast with controlled factory settings, field harvesting environments impose stringent constraints on power consumption and physical space while simultaneously being characterized by complex electromagnetic interference and high-frequency mechanical vibrations. Under these rigorous conditions, traditional industrial computers relying on general-purpose CPUs which often exhibit nondeterministic system latency due to their serial processing mechanisms, resulting in spatiotemporal misalignment between detection and pneumatic actuation. Consequently, the technological trajectory is shifting towards heterogeneous computing paradigms. This approach leverages the massive parallelism of FPGAs for high-throughput data preprocessing, while utilizing embedded GPUs to accelerate deep learning inference. Such architecture not only minimizes inference latency to the millisecond scale via model pruning and quantization but also facilitates nanosecond-level precise triggering at the hardware layer to compensate for vibration-induced transmission errors. Furthermore, to address dust-laden environments, edge-deployed adaptive algorithms must possess the capability for real-time lens contamination detection and image enhancement, thereby ensuring system robustness under harsh operational conditions.

Furthermore, the airborne sorting module must possess extremely high environmental adaptability and system integration. Facing high-concentration dust invasion and diurnal temperature variations in the field, the embedded sorting module needs to meet industrial-grade protection standards, equipped with automatic air knives for dust removal and constant-temperature heat dissipation systems to prevent lens dust accumulation and thermal failure of core components. At the system integration level, the sorting module needs to be deeply interconnected with the harvester’s CAN bus control system, dynamically adjusting the sorting belt speed and pneumatic rejection pressure in real time according to the harvester’s travel speed and header feed rate, achieving adaptive synergy of agricultural machinery and agronomic parameters.

In summary, field in situ sorting technology is a deep intersection of mechanical engineering, photoelectric sensing, edge computing, and control theory; its breakthrough will thoroughly reshape the harvesting form of the processing tomato industry, driving agricultural mechanization toward a higher stage of digitalization and intelligence.

Given the significant differences in principles and application scenarios of the aforementioned technologies, a comprehensive trade-off between multiple performance indicators is essential when developing next-generation intelligent harvesting equipment. To provide a clearer evaluation of their practical efficacy in unstructured field environments, Table 4 systematically compares visible imaging, NIR, X-ray, CNN-based vision, and compliant actuation across several critical dimensions, including effective conditions, limitations, throughput, robustness, cost, computing demand, and suitability for field deployment. This comparative synthesis highlights the inherent trade-offs between different technological approaches, aiming to provide a strategic reference for selecting optimal integrated solutions tailored to specific operational constraints, such as the high-dust and variable lighting conditions of open-field tomato harvesting.

### 6.3. Intelligence, Adaptive Control, and Practical Constraints

The remote operation and maintenance (O&M) system based on cloud data serves as a critical means to resolve the industry pain points of the Xinjiang production region’s vast geographic expanse and the long response cycles for after-sales service [213,214]. By integrating industrial-grade wireless communication modules, modern color sorters can upload equipment operational status data, fault codes, and life indicators of key components to cloud servers in real time. At the cloud level, big data analysis platforms utilize feature data uploaded via edge computing to construct digital twin models of the equipment. Through the deep mining of massive historical data, the system can achieve predictive maintenance for faults. Specifically, before physical failure occurs in core components such as pneumatic nozzles, solenoid valves, or light sources, early warnings are issued based on the degradation trends of their performance curves; this guides maintenance personnel to perform precise preventive replacement, thereby maximizing the reduction in production interruptions and raw material spoilage caused by sudden downtime.

Furthermore, remote collaboration functions based on cloud architecture endow the equipment with the capability for continuous evolution. R&D personnel need not visit remote processing plants or farmlands in person; they can access the equipment control core via remote desktops to view real-time image data and waveform logs, performing deep fault diagnosis and parameter fine-tuning. More importantly, with the continuous iteration of deep learning algorithms, the cloud platform can push the latest recognition models and control firmware on terminal devices via Over-the-Air (OTA) technology. This implies that color sorting across different geographic locations and processing of different tomato varieties can share the latest algorithmic achievements in real time, realizing full lifecycle management of algorithm models and continuous leaps in performance. This intelligent architecture of edge–cloud synergy not only significantly reduces the O&M costs for enterprises but also lays a solid technical foundation for realizing the vision of unmanned factories in the tomato processing industry [215,216,217].

To overcome the aforementioned challenges and further elevate the impact of this research domain, future investigations should prioritize two critical directions. First, a synthesis of the current literature reveals a significant gap in the durability profiling of advanced materials. While flexible sorting mechanisms demonstrate reduced damage rates in controlled environments, empirical evidence regarding the long-term fatigue resistance of TPU- and silicone-based soft robotic end-effectors under abrasive field conditions remains scarce, highlighting a critical area for subsequent validation. Second, field trial data highlighted in recent studies reveal that edge AI power constraints constitute a primary operational bottleneck. Existing research shows that maintaining real-time processing capabilities under the severe power limitations of mobile harvesters requires the targeted integration of power-efficient Neural Processing Units (NPUs). Tailoring NPUs specifically for in-field equipment will provide the optimal balance between maintaining the high-throughput inference required for visual sorting and adhering to the stringent low-power limitations of tractor alternators.

Furthermore, while X-ray technology offers promising non-destructive internal defect detection, its application in open-field environments encounters significant safety and regulatory hurdles. Ensuring adequate radiation shielding on a high-vibration, mobile harvester to protect operators and field workers is technically challenging and subjected to strict regulatory compliance, which further complicates its commercial adoption. Finally, during the extremely short harvesting window, overcoming the slower dynamic response limits of soft materials to balance the engineering trade-off between high throughput and high-speed “zero-damage” sorting remains a critical hurdle that must be addressed before practical implementation.

## Figures and Tables

**Table 3:** Comparative framework of sensing technologies for tomato harvesters.

Sensing Modality	Detectable Defect Type	External vs. Internal Capability	Robustness to Dust & Vibration	Vibration Throughput	Computational Burden	Risk of Fruit Damage	Readiness for Harvester-Mounted Deployment
Visible Imaging (CCD/CMOS + CNN)	Green fruits, surface mold, stem/leaves, color defects	External only	Low	Very High	High	None (non-contact)	High (currently the industry standard for bulk sorting)
Near-Infrared (NIR) Spectroscopy	Internal rot, moisture variations, invisible bruises	Both	Medium	Medium–High	Medium	None (non-contact)	Medium (emerging in field, limited by vibration stability)
X-ray Transmission	High-density impurities (stones, dense clods, glass)	Internal & External	High	Medium	High	None (non-contact)	Low (hindered by bulky hardware and radiation safety constraints)
Tactile/Force Sensing	Overripe softening, liquefied interiors (soft rot)	Internal	High	Low–Medium	Low	Low–Medium (mitigated by soft robotic effectors)	Low (mostly experimental; struggles with high-speed material flow)

**Table 4:** Comparative analysis and trade-offs of sensing and sorting technologies for mechanized tomato grading.

Technology	Effective Conditions	Key Limitations	Throughput	Robustness (Field)	Cost	Computing Demand	Field Deployment Suitability
Visible Imaging (RGB)	Clean environments, clear color differences (e.g., red vs. green).	Fails with metameric objects (red soil/red fruit); highly sensitive to dust and lighting changes.	Very High	Low to Medium	Low	Low to Medium	High (standard baseline, but requires shielding)
NIR Spectroscopy	Detecting internal defects, moisture content, and differentiating soil from fruit.	Sensitive to surface moisture/dirt; lower spatial resolution than RGB.	Medium	Medium	Medium to High	Medium	Medium (requires controlled lighting/calibration)
X-ray Transmission	Detecting dense foreign objects (stones, clods, metal) wrapped in mud/slurry.	Radiation safety concerns; bulky equipment; cannot detect color defects.	High	High	Very High	High	Low (difficult to integrate safely on mobile harvesters)
CNN-based Vision (e.g., YOLO)	Complex backgrounds, high occlusion, variable lighting, overlapping fruits.	Requires massive, annotated datasets; susceptible to performance drops if field conditions change drastically.	High (Requires GPU)	High (Algorithmically)	Medium	Very High	Medium to High (requires edge-computing hardware like FPGAs/GPUs)
Compliant Actuation (Soft Robotics)	Handling easily bruised, delicate, or overripe tomatoes.	Slower response time than air jets; flexible materials are prone to wear/tear from hard stones/vines.	Low to Medium	Medium	Medium	Low	Medium (durability is a major bottleneck in harsh field conditions)

## Data Availability

The data used to support the findings of this study are available from the corresponding author upon request.
